# Roles of Inflammasomes in Epstein–Barr Virus-Associated Nasopharyngeal Cancer

**DOI:** 10.3390/cancers13081786

**Published:** 2021-04-08

**Authors:** Chin King Looi, Ling-Wei Hii, Felicia Fei-Lei Chung, Chun-Wai Mai, Wei-Meng Lim, Chee-Onn Leong

**Affiliations:** 1 School of Postgraduate Studies, International Medical University, Kuala Lumpur 57000, Malaysia; 00000028295@student.imu.edu.my (C.K.L.); lingweihii@imu.edu.my (L.-W.H.); 2 Center for Cancer and Stem Cell Research, Institute for Research, Development and Innovation (IRDI), International Medical University, Kuala Lumpur 57000, Malaysia; chunwai_mai@imu.edu.my (C.-W.M.); weimeng_lim@imu.edu.my (W.-M.L.); 3 School of Pharmacy, International Medical University, Kuala Lumpur 57000, Malaysia; 4 Mechanisms of Carcinogenesis Section (MCA), Epigenetics Group (EGE), International Agency for Research on Cancer World Health Organisation, CEDEX 08 Lyon, France; chungf@fellows.iarc.fr; 5 State Key Laboratory of Oncogenes and Related Genes, Renji-Med X Clinical Stem Cell Research Center, Ren Ji Hospital, School of Medicine, Shanghai Jiao Tong University, Shanghai 200127, China

**Keywords:** inflammasome, nasopharyngeal carcinoma, Epstein–Barr virus, inflammation, cancer, viral evasion, immune response

## Abstract

**Simple Summary:**

Inflammasomes play a key role in mediating innate immunity by regulating the processing and production of pro-inflammatory cytokines and eliminating foreign pathogens via pyroptosis. The expression of Epstein–Barr virus (EBV) viral antigens during EBV infection, a known causative agent of nasopharyngeal carcinoma (NPC), can trigger host’s antiviral immune response through activation of the inflammasomes and subsequent production of pro-inflammatory cytokines. This review explores the roles of inflammasomes during viral infection, the possible impact of inflammasomes on oncogenesis in EBV-associated NPC, and current developments in targeting inflammasomes for cancer treatment. With the contrasting roles of inflammasomes reported in different types of cancers, this paper aims to inspire further investigations into the exact role and mechanism of inflammasomes in EBV-associated NPC, as well as the therapeutic potentials of targeting inflammasomes in NPC.

**Abstract:**

Epstein–Barr virus (EBV) infection is recognised as one of the causative agents in most nasopharyngeal carcinoma (NPC) cases. Expression of EBV viral antigens can induce host’s antiviral immune response by activating the inflammasomes to produce pro-inflammatory cytokines, such as interleukin-1β (IL-1β) and IL-18. These cytokines are known to be detrimental to a wide range of virus-infected cells, in which they can activate an inflammatory cell death program, called pyroptosis. However, aberrant inflammasome activation and production of its downstream cytokines lead to chronic inflammation that may contribute to various diseases, including NPC. In this review, we summarise the roles of inflammasomes during viral infection, how EBV evades inflammasome-mediated immune response, and progress into tumourigenesis. The contrasting roles of inflammasomes in cancer, as well as the current therapeutic approaches used in targeting inflammasomes, are also discussed in this review. While the inflammasomes appear to have dual roles in carcinogenesis, there are still many questions that remain unanswered. In particular, the exact molecular mechanism responsible for the regulation of the inflammasomes during carcinogenesis of EBV-associated NPC has not been explored thoroughly. Furthermore, the current practical application of inflammasome inhibitors is limited to specific tumour types, hence, further studies are warranted to discover the potential of targeting the inflammasomes for the treatment of NPC.

## 1. Introduction

An estimated 15.4% of worldwide human cancers are attributable to carcinogenic infections [1]. The Epstein–Barr virus (EBV) is a human herpesvirus that has been classified as a Group 1 carcinogen by the International Agency for Research on Cancer (IARC) in view of its association with certain lymphoid malignancies, including Burkitt lymphoma (BL), Hodgkin’s lymphoma (HL), immunosuppression-related non-Hodgkin’s lymphoma (NHL), and cancer of the nasopharynx [2]. Up to 40% of Hodgkin’s lymphoma cases are EBV positive. HL in males, children, older adults, and in developing countries are slightly more likely to develop EBV-associated HL, while the onset of nodular sclerosing HL (NSHL) in young adults and industrialised countries is typically EBV-negative [3]. BL is an aggressive B cell malignancy which can be classified into three variants: endemic, sporadic and immunodeficiency-associated. Among the endemic BL cases, over 95% are associated with EBV and is prevalent in equatorial belt of Africa and other parts of the world where malaria is hyperendemic [4]. Nasopharyngeal carcinoma (NPC) is a cancer originating from the chronic inflammation of the nasopharyngeal epithelium [5], nearly 100% of which are closely associated with EBV infection [6,7]. EBV expresses a series of latent viral genes that support the growth of EBV-infected NPC cells. These genes include latent membrane proteins (LMP1 and LMP2), Epstein–Barr nuclear antigen (EBNA1), BamH1 A fragment rightward reading frame 1 (BARF1), non-polyadenylated and non-protein coding small EBV RNAs (EBER1 and EBER2), BamHI A rightward transcripts (BARTs) and BART microRNAs (miR-BARTs) [8].

NPC has been identified as one of the most common head and neck cancers in parts of Southeast Asia, Southern China, and North Africa, with 5-year survival rates of 70–80% [9,10]. Due to its complex anatomic location and high radiosensitivity, radiotherapy (RT) is highly recommended in the treatment of non-metastatic or stage I NPC [11]. However, the majority of patients (80%) are diagnosed at advanced stages, as early stage NPC presents with innocuous symptoms, such as headache, nose bleed, nasal obstruction and nasal discharge [9,10]. Hence, more intense treatment, such as combined chemoradiotherapy, has been recommended by the European Society for Medical Oncology (ESMO) Clinical Practice Guidelines and National Comprehensive Cancer Network (NCCN) Clinical Practice Guidelines in Oncology (Version 2.2020) to treat NPC patients with stage II and higher cancers due to the improved survival benefits [12,13]. For example, a phase III clinical trial that involved 230 stage II NPC patients reported that combination of RT with concurrent weekly cisplatin (30 mg/m^2^) resulted in significantly better clinical outcomes compared to RT alone, conferring survival benefits to stage II NPC patients [14]. However, the treatment outcomes remain unsatisfactory as patients may encounter unwanted adverse effects, disease recurrence, and development of distant metastasis [14,15,16]. Therefore, a novel treatment approach is needed to improve therapeutic outcomes.

Inflammation is a protective immune response mounted by the host’s innate immune system, allowing the host to defend against viral, bacterial, fungal infections, or internal insults. However, sustained inflammation may cause excessive tissue damage, leading to the development of autoimmune diseases and chronic inflammatory diseases [17,18]. In addition, accumulating evidence demonstrates that the inflammatory microenvironment is a hallmark of carcinogenesis, promoting tumour initiation, and development through cytokine-induced stimulation of malignant cells [19]. Chronic inflammation is known to promote carcinogenesis by inducing expression of growth factors, resistance to growth inhibition, and apoptosis, as well as initiating tumour development via angiogenesis and metastasis [20]. More importantly, aberrant tumour-promoting inflammation could contribute to tumour cell evasion from immune surveillance by facilitating the infiltration of immune suppressor cells, such as myeloid-derived suppressor cells (MDSCs) and tumour-associated macrophages (TAMs) [21,22].

The inflammasome is an intracellular multi-protein complex that is important in initiating inflammatory immune response via activation of inflammatory caspases, particularly caspase-1. As a result, it is a key player in regulating acute as well as chronic inflammation [23]. It provokes a high production of pro-inflammatory cytokines such as interleukin (IL)-1β and IL-18 in response to the danger signals in tumour microenvironment (TME), resulting in the activation of a pro-inflammatory immune response [23,24,25]. However, its functional roles in tumour progression remains poorly understood. Multiple studies have indicated that the inflammasome is a double-edged sword in cancers, as it can act as a tumour suppressor to protect against tumourigenesis, or act as a tumour promoter to facilitate tumour progression and survival [26].

Pattern recognition receptors (PRRs) are key players in initiating the host immune defence system via the recognition of pathogen- or danger-associated molecular patterns (PAMPs or DAMPs) [25]. Stimulation of the PRRs by intracellular host stress or tissue damage will lead to the activation, maturation, and production of pro-inflammatory cytokines followed by the adaptive immune responses [27,28]. PRRs induce two different transcription factor-mediated pathways, namely the canonical and non-canonical nuclear factor kappa B (NF-κB) pathways, upon interaction with their corresponding ligands [29]. Canonical NF-κB induces the secretion of IL-6 and tumour necrosis factor (TNF)-α, as well as the priming and activation of inflammasomes. On the other hand, the non-canonical NF-κB, will be driven by the interferon regulatory factor 3 (IRF3) pathway to release type I interferon (IFN), which leads to the activation of Janus kinase-signal transducer and activator of transcription (JAK-STAT) signalling and expression of IFN-stimulated genes [29].

**Figure 1:**
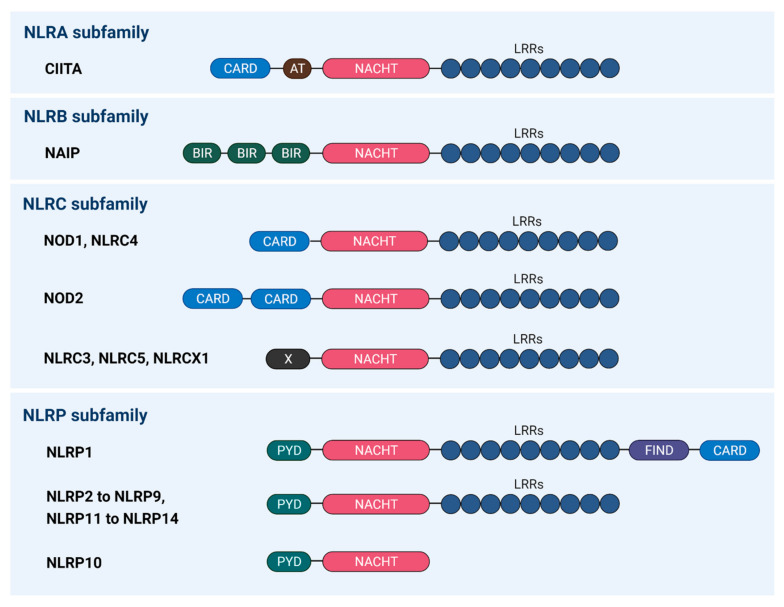
Human NLR subfamilies and their members. The canonical structure of the NLR consists of a tripartite structure, including a N-terminal domain, a central NACHT domain, and C-terminal LRRs. A total of 22 human NLRs have been identified to date and they can be classified into four subfamilies based on their N-terminal effector domains. NLRA subfamily consists of one NLR member–CIITA, which possesses AT at its N-terminal region, while NLRB subfamily comprises NAIP that have BIR domains. NLRC subfamily members are characterised with the presence of CARD domain; NLRP subfamily members are PYD-containing NLRs and they are well-known for the regulation of NF-κB pathway to modulate the release of pro-inflammatory cytokines through the formation of the oligomeric inflammatory complex termed the “inflammasome”. (NLR, NOD-like receptor; NACHT, also known as NOD (nucleotide binding and oligomerisation domain); LRRs, leucine-rich repeats; AT, acidic transactivation domain; PYD, pyrin domain; CARD, caspase activation and recruitment domain; BIR, baculoviral inhibition of apoptosis protein repeat; NF-κB, nuclear factor kappa B; FIND, function-to-find domain; X, unknown).

The nucleotide-binding oligomerisation domain-like receptors, or NOD-like receptors (NLRs) (also known as nucleotide-binding leucine-rich repeat receptors), are an important subfamily of the host PRRs that have the ability to recognise PAMPs and DAMPs to initiate the innate immune response [25]. The NLR family comprises 22 members which can be classified into four subfamilies, the NLRA, NLRB, NLRC, and NLRP, based on their N-terminal effector domain structures (Figure 1) [30]. For example, NLRA has an acidic transactivation domain; NLRB contains a baculoviral inhibition of apoptosis protein repeat (BIR) domain; NLRC has a caspase-recruitment and activation domain (CARD); and NLRP has a pyrin domain (PYD) [30]. Several findings focused on NLRs demonstrate that they can form inflammasomes that mediate the inflammatory signalling. Various inflammasomes have been discovered since year 2002 [23,31] and can be classified into two types: canonical and non-canonical [26]. Canonical inflammasome activation is caspase-1 dependent. It consists of an NLR, apoptosis-associated speck-like protein (ASC), and caspase-1 [32]. The ASC serves as an adaptor protein and is composed of two death-fold domains: PYD and CARD. The PYD and CARD domains allow for interactions between NLR and caspase-1 or caspase-11, with or without co-binding of ASC for inflammasome assembly [30,33]. In contrast, non-canonical inflammasome activation is dependent on caspase-11 (mice) or caspase-4/5 (humans). Direct recognition of the cytosolic lipopolysaccharide (LPS) by caspase-4/5 (caspase-11 in mice) forms LPS–caspase-4/5 complexes which are then oligomerised to form caspase-4/5 or caspase-11 non-canonical inflammasome via CARD domain [34]. Activated caspase-4/5 or caspase-11 plays a key role in triggering the activation of pyroptosis [34,35,36].

As components of the inflammasomes, NOD-like receptor pyrin domain containing 1 and 3 (NLRP1 and NLRP3) are important in mediating host immune response against viral infections by initiating inflammation. However, studies have demonstrated that viruses have evolved mechanisms to evade inflammasome-mediated antiviral immune response [37,38]. In this review, we highlight the functional roles of inflammasomes in EBV-associated NPC and viral evasion mechanisms. We also discuss the therapeutic potential of targeting the inflammasomes in cancer immunotherapy.

## 2. Roles of Inflammasomes during Viral Infection

**Figure 2:**
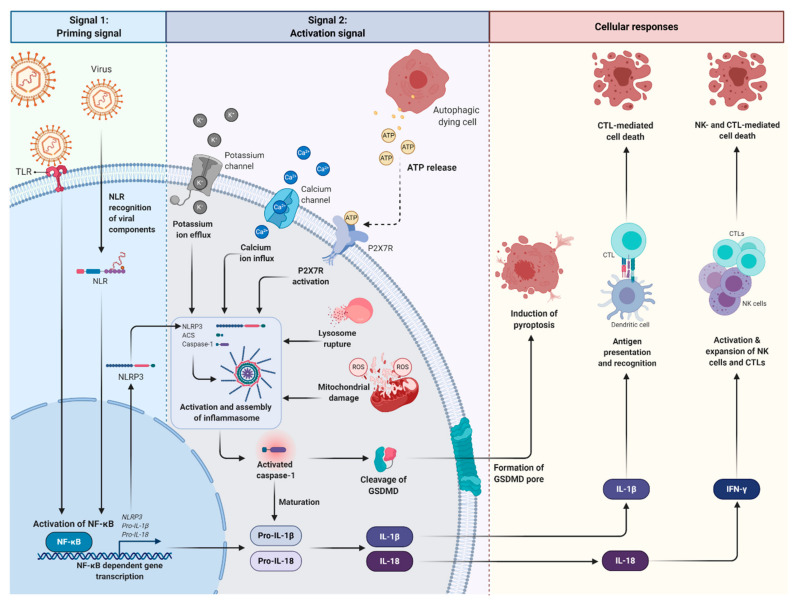
An overview of canonical inflammasome activation during Epstein–Barr virus (EBV) infection. The viral constituents can serve as a PAMP and be recognised by PRRs such as TLR and NLR. As such, TLR/NLR can recognise EBV during infection. The interaction of viral components with TLRs or NLRs results in the transcription and expression of the NLRP3 inflammasome, pro-IL-1β and pro-IL-18 via NF-κB activation (signal 1). An additional stimulus (signal 2) induced by cytosolic danger signals (referred to as DAMPs), such as lysosomal damage, mitochondrial ROS, potassium efflux and calcium ion influx, is usually required for the production and subsequent extracellular release of IL-1β and IL-18. Moreover, the binding of ATP released from autophagic dying cells to P2X7R induces P2X7R activation and subsequent potassium ion efflux and calcium ion influx. Together, these signals promote assembly of the NLRP3 inflammasome to ASC which then recruits pro-caspase-1 via its CARD. This leads to the oligomerisation and activation of the inflammasome complex. The inflammasome complex triggers the cleavage of pro-caspase-1 into caspase-1, and subsequent maturation of pro-inflammatory cytokines. Activated caspase-1 induces pyroptosis of infected cells via cleavage of GSDMD. On the other hand, IL-1β and IL-18 are important mediators of innate and adaptive immune response. (PAMP, pathogen-associated molecular pattern; TLRs, toll-like receptors; NLRs, NOD-like receptors; IL-1β, interleukin-1β; IL-18, interleukin-18; NF-κB, nuclear factor kappa B; DAMPs, damage-associated molecular patterns; ROS, reactive oxygen species; ATP, adenosine triphosphate; P2X7R, P2X7 purinergic receptor; NLRP3, NOD-like receptor pyrin domain containing 3; ASC, apoptosis-associated speck-like protein; CARD, caspase activation and recruitment domain; GSDMD, gasdermin D).

Canonical inflammasome activation is essential in triggering host antiviral immune responses (Figure 2) [39,40]. In the rest state, inflammasomes are expressed at low level to prevent aberrant assembly and activation of inflammasomes. Inflammasomes can be activated in response to the invading viruses and cytosolic danger signals, such as maturation of lysosome, mitochondrial damage, aberrant ion concentrations, and accumulation of misfolded protein aggregates [29,41].

Inflammasomes require two signals to become functionally activated: a priming signal (signal 1) and an activation signal (signal 2) [42]. Priming signals are initiated by the binding of viral components to PRRs such as the toll-like receptor (TLR) or NLR ligands which activate NF-κB. Activation of NF-κB promotes the transcription and expression of the inflammasome, pro-IL-1β and -IL-18, which, after translocation from the nucleus to the cytosol, remain inactive until stimulated by the second signal [24,40,42]. These priming signals also promote post-translational modifications of inflammasome components, including inflammasome deubiquitination, ubiquitination, and phosphorylation of ASC, which is required for subsequent activation and assembly of the inflammasome complex [43]. The second activation signal is required for the inflammasome assembly. It is triggered by PAMPs or DAMPs, including hypoxia, extracellular adenosine triphosphate (ATP), or reactive oxygen species (ROS), which cause potassium and calcium ion fluxes, lysosomal damage, mitochondrial ROS production, ATP release and subsequent activation of the ATP gate ion channel, P2X7 purinergic receptor (P2X7R) [42,44]. Activation of P2X7R by ATP increases the pore permeability of membrane receptors, triggering potassium ion efflux and calcium ion influx, which are crucial for inflammasome assembly and caspase-1 activation [45,46]. Non-transcriptional priming of NLRP3 inflammasome for inflammasome activation can also occur through deubiquitination, in a process dependent on mitochondrial ROS (mtROS) and ATP [47].

The NLRP3 inflammasome is then assembled with ASC, encoded by the *Pycard* gene and recruits pro-caspase-1 through its CARD domain, resulting in the oligomerisation and activation of the inflammasome complex [29]. Of note, the assembly of the NLRP1 inflammasome complex is independent of ASC [26] as it contains a C-terminal CARD domain which can activate caspase-1 directly [48]. The inflammasome complex acts as a caspase-1 activator, triggering the cleavage of pro-caspase-1. Activated caspase-1 is required for the activation of IL-1β and IL-18.

Cytokines are important promoters of inflammation and activation of adaptive immune response via NF-κB and mitogen-activated protein kinase (MAPK) signalling [49,50]. The suppression of IL-1β and IL-18 production in ASC-deficient mice has clearly indicated the importance of ASC in caspase-1 activation followed by the release of downstream cytokines [51]. IL-1β drives the polarisation of T helper cells (CD4^+^ T cells), foreign antigen recognition, induces the activation and differentiation of antigen-specific cytotoxic T lymphocytes (CTLs) [52,53,54]. More importantly, it has been shown to significantly inhibit tumour growth and recurrence as compared to pro-IL-1β, indicating the importance of inflammasome activation in promoting the production of IL-1β for tumour suppression [44]. On the other hand, IL-18 is necessary for driving natural killer (NK) cell as well as CTL activity through the promotion of IFN-γ production [24,49].

Caspase-1 also triggers programmed cell death, such as pyroptosis and necroptosis [45]. Unlike apoptosis, pyroptosis is initiated by the cleavage of pro-pyroptotic factor, the gasdermin D (GSDMD), which targets and permeabilises cellular membrane by inducing the formation of pores in the plasma membrane, compromising its integrity [55,56]. Consequently, the resulting imbalance causes osmotic swelling and osmotic lysis [57]. Cellular components released during pyroptotic cell death, including IL-1 and ATP, in turn promote the recruitment of neutrophils to the inflammatory site to aid in the eradication of invading viruses [29,45,57]. As a result, inflammasome activation is essential for antiviral immunity and inflammatory response.

## 3. Virus Evasion of Inflammasome-Mediated Immune Response

CTLs are critical soldiers of adaptive immune response. In order to become functionally activated and effective in eliminating infected/tumour cells, CTLs require three signals; interaction of T cell receptor (TCR) with major histocompatibility complex (MHC)-peptide complex, CD28/CD80 co-stimulatory signal, and inflammatory cytokine signals [54,58]. Recent studies have reported that foreign antigen presentation and perforin released from CTLs can serve as a priming signal and are required for the subsequent activation of NLRP3 inflammasome complexes and production of IL-1β [59,60,61]. Perforin forms pores on target cell membrane and induces calcium ion influx into the cytosol, which triggers inflammasome activation. In vivo, the loss of NLRP3 inflammasome or caspase-1 activity has been found to inhibit the priming and activation of CTLs, thereby significantly blocking the cytotoxic effects of CTLs against tumour cells in mouse models [50,59]. As a result, these findings clearly indicate the critical role of inflammasomes in mediating innate and adaptive immune response against tumour cells via secretion of inflammatory cytokines. Here, we have identified several possible strategies used by viruses to counteract or evade inflammasome-mediated immune response: (a) inhibition of inflammasome assembly; (b) degradation of inflammasomes; (c) alteration of NF-κB signalling; (d) modulating the production of pro-inflammatory cytokines.

### 3.1. Inhibition of Inflammasome Assembly and Activation

The assembly and activation of inflammasomes can be directly or indirectly inhibited by viruses, hence suppressing antiviral immunity while facilitating sustained viral replication. This is best illustrated by the action of Kaposi’s sarcoma-associated herpesvirus (KSHV) Orf63. KSHV Orf63 protein is the viral NLRP1 homolog which lacks the PYD and CARD domains [62]. It interacts with the human NLRP1 inflammasome to prevent oligomerisation and assembly of the NLRP1 inflammasome with pro-caspase-1. As a result, caspase-1 activity and subsequent secretion of IL-1β and IL-18 are inhibited, demonstrating that viral genome-encoded protein products act as inhibitory factors of inflammasome activation [62]. Paramyxovirus V proteins have been shown to directly interact with the NLRP3 inflammasome, inhibiting NLRP3 self-oligomerisation and subsequent recruitment of ASC to prevent NLRP3-ASC oligomerisation. This molecular event inhibits inflammasome activation as well as IL-1β production [63].

In addition, some viruses also encode proteins which are homolog to PYD-only proteins (POPs) to prevent the recruitment and oligomerisation of inflammasome adaptor proteins such as ASC, in turn blocking the formation of a functional inflammasome complex. POPs are known as the negative regulator of inflammasome assembly as well as caspase-1-dependent pro-inflammatory cytokines secretion [64,65]. POP1 can bind to ASC and interfere with the recruitment and oligomerisation of ASC to activated PYD–NLR, resulting in the inhibition of inflammasome activation and suppressed NF-κB activity, thus facilitating immune evasion [65,66]. Similarly to POP1, POP2 interacts with ASC, disrupting the NLRP3 inflammasome structure, thus suppressing caspase-1 activation and cytokine production [66,67]. For example, poxviruses-encoded viral POP has been recognised as a novel immune evasion protein that impairs host immune response by binding with ASC, hence blocking ASC-inflammasome interaction [68]. This event leads to the inhibition of inflammasome complex-mediated activation of pro-caspase-1 and subsequent release of IL-1β and IL-18.

Cellular processes such as potassium ion efflux, calcium ion flux and production of ROS are also known to affect the assembly and activation of the inflammasome complex [24]. Indeed, the blockade of ROS production and treatment with potassium chloride (KCl) upon Mayaro virus (MAYV) infection have resulted in a dose-dependent reduction of IL-1β secretion [69]. Another similar study indicated that the ROS production in EBV-associated NPC cells can activate the NLRP3 inflammasome and enhance antitumour immunity via IL-1β production [44]. These findings clearly indicate the importance of ROS production and potassium ion efflux in NLRP3 inflammasome activation in response to viral infection. Moreover, myeloid-specific microRNA miR-223 has been identified as another important regulator of inflammasome activity. It downregulates NLRP3 expression and inhibits IL-1β production by binding to the 3′ untranslated region (UTR) of the NLRP3 inflammasome [70]. EBV miR-BART15 was shown to specifically target the same site as miR-223 on the 3′ UTR of the NLRP3 inflammasome and reduce NLRP3 expression. Similarly, miR-BART15 can also be secreted by infected B cells through exosomes and suppressed inflammasome activity in non-infected cells, and hence further amplifying the immunosuppressive state [71].

### 3.2. Degradation of Inflammasome and Its Components

Ubiquitination and degradation of the NLRP3 inflammasome is critically important in regulating inflammasome activation and its biological function. E3 ligase is identified as a negative regulator of the inflammasome, by controlling its protein expression levels through proteasome activity as well as autophagy [72]. Although some studies have shown that inflammasome complexes exhibit increased levels of ubiquitination upon stimulation [47,73,74], it has also been reported that the inflammasome itself is deubiquitinated in response to the activation signal, allowing for the activation of inflammasome [73,74,75,76]. Hence, whether ubiquitination is required for activation of inflammasome remained controversy.

Degradation of herpes simplex virus (HSV)-1 capsid proteins by cellular ubiquitin proteasome machinery unmasks viral DNA for inflammasome recognition and activation [77]. However, constitutive ubiquitination of the inflammasome may increase proteasomal degradation of the inflammasome, in turn inhibiting the formation of active inflammasomes [72,76,78].

The C protein of human parainfluenza virus type 3 (HPIV3) can antagonise inflammasome activation by triggering ubiquitin-mediated NLRP3 protein degradation [79]. Pro-IL-1β has also been shown to be negatively regulated by ligase-mediated ubiquitination in human papillomaviruses (HPVs)-positive cells, which ultimately attenuates IL-1β production in virus-infected cells [80]. In addition, enterovirus 71 (EV71) secretes viral proteases 2A and 3C to cleave NLRP3 protein or directly interact with NLRP3 to inhibit IL-1β secretion [78]. The cleavage of GSDMD at Q193 and G194 amino acid residues by the EV71 viral protease 3C leads to the formation of non-functional GSDMD fragments [81]. Ultimately, it results in enhanced viral replication and reveals a new mechanism of viral evasion. Hence, dysregulation of ubiquitination in virus-infected cells and the subsequent degradation of the inflammasome are apparently a novel mechanism for viral immune evasion and a key step for the initiation of carcinogenesis.

### 3.3. Dysregulation of NF-κB Signalling

NF-κB is a family of five inducible transcription factors, including NF-κB1/p50, NF-κB2/p52, RelA/p65, RelB, and c-Rel. It acts as a master regulator of inflammatory response, which regulates immune functions and transcriptional induction of pro-inflammatory cytokines, highlighting its role in antitumoural immunity [17]. Several findings have shown that the NF-κB signalling pathway is important in regulating inflammasome activity. It mediates the priming signal of inflammasome activation by triggering the transcription of inflammasome genes upon stimulation by cytokines and PRRs [17,82,83]. Hence, dysregulation of NF-κB signalling in inflammasome activation may lead to chronic inflammation and set the stage for cancer development. Chronic inflammation drives the recruitment of MDSCs which promotes NF-κB-induced Tregs to stimulate angiogenesis and evasion of immune surveillance [84].

**Figure 3:**
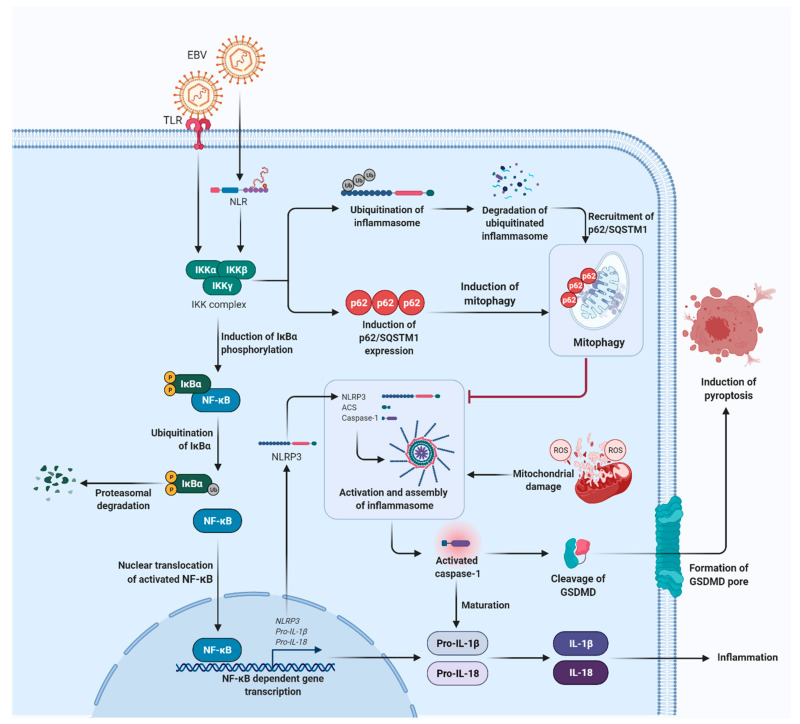
IKKs act as the negative regulator of inflammasome activation. IKK kinase complex which is made up of two kinases (IKKα and IKKβ) and a regulatory subunit (IKKγ), is the key regulator of NF-κB signalling. The phosphorylation of the inhibitor of NF-κB proteins, particularly the IκBα by IKKs, resulting in the ubiquitination and proteasomal degradation of IκBα. The activated NF-κB is then translocated into the nucleus to regulate target gene expression, which contributes to inflammasome activation, maturation and production of IL-1β and IL-18. The ROS released from damaged mitochondria serve as an important inflammasome activating signal, however, IKKs can induce autophagy clearance of damaged mitochondria by inducing the expression and accumulation of autophagy receptor, p62/SQSTM1. In addition, degradation of inflammasome components through ubiquitination triggers expression of p62/SQSTM1, and hence, negatively regulates inflammasome activation by promoting mitophagy. (IKK, inhibitory kappa B kinases; NF-κB, nuclear factor kappa B; IκBα, inhibitory subunit of nuclear factor kappa B alpha; ROS, reactive oxygen species; p62/SQSTM1, p62/sequestosome-1).

The inhibitory kappa B kinases (IKKs) with two catalytic subunits, IKKα and IKKβ, and a regulatory subunit named NF-κB essential modulator (NEMO) or IKKγ are identified as the key regulators of the NF-κB cascade [85]. Upon activation, IKK complex phosphorylates the inhibitor of NF-κB proteins, particularly the inhibitory subunit of nuclear factor kappa B alpha (IκBα). This in turn triggers ubiquitination and proteasomal degradation of IκBα, resulting in nuclear translocation of activated NF-κB to regulate target gene expression [17,86]. A recent study has reported on the inhibitory role of IKKs in inflammasome activation and it is postulated that IKKs could suppress inflammasome activation in EBV-infected cells, and thereby, circumventing innate immune response (Figure 3) [82]. IKKs were shown to suppress inflammasome activation by inducing the expression of an autophagy receptor, the sequestosome-1 (SQSTM1), which encodes p62 (p62/SQSTM1) [82]. The autophagy receptor recruits damaged mitochondria for autophagic clearance, hence, preventing the release of mtROS and mitochondrial DNA (mtDNA), which act as inflammasome activators [82,83,87]. NF-κB can also negatively regulate inflammasome activation by degrading major components of the inflammasome complex via inflammasome ubiquitination. As a consequence, this leads to the recruitment of p62/SQSTM1 [88]. Conversely, downregulation of p62/SQSTM1 expression or blockade of autophagy have been shown to increase ROS production from damaged mitochondria, promote NLRP3 activation, and augment IL-1β secretion [88].

**Figure 4:**
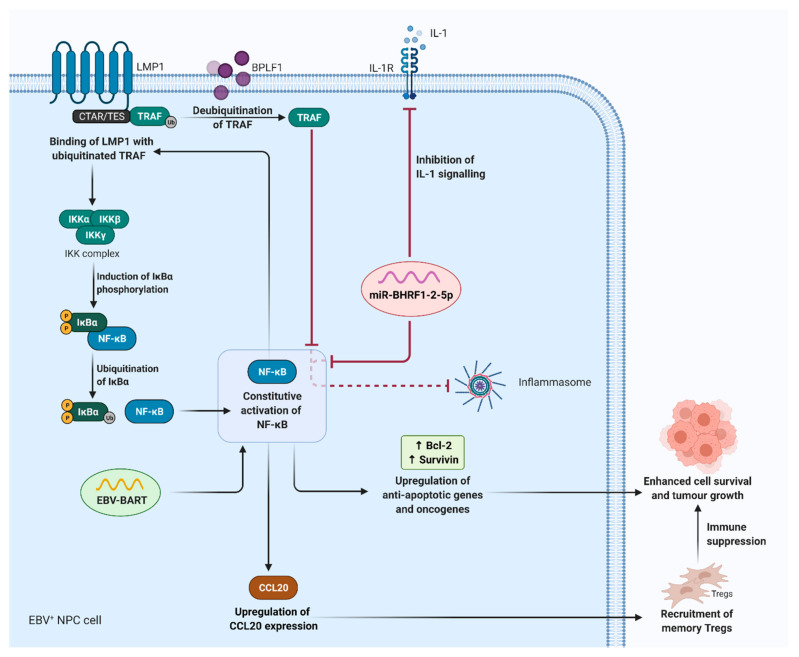
Overview of the role of EBV genes in regulating NF-κB activation. LMP1 induces a constitutive activation of NF-κB via interaction of CTAR/TES with ubiquitinated TRAFs, followed by phosphorylation of the IκBα to sustain latent infection. Meanwhile, NF-κB upregulates LMP1 expression, creating an amplification loop that displays constitutive NF-κB activity during EBV latency. Dysregulation of NF-κB activity may lead to unfavourable immune response such as recruitment of Tregs via upregulation of CCL20 expression. This in turn diminishes cytotoxicity effect of CTLs and results in the suppression of immune surveillance. In addition, constitutive NF-κB activity contributes to the malignant progression of EBV-associated NPC through the upregulation of genes involved in proliferation, anti-apoptosis, and maintaining latent infection. An upregulation of EBV BART is also associated with constitutive activation of NF-κB signalling in NPC cells. On the other hand, EBV may inhibit the activation of NF-κB and the subsequent pro-inflammatory cytokines production. For instance, the expression of BPLF1, an EBV deubiquitinase can deubiquitinate TRAF to antagonise NF-κB activity as well as inflammasome activation; miR-BHRF1-2-5p as EBV miRNA, can directly target the 3′UTR of the IL-1R, and thereby, inhibiting IL-1 signalling. (EBV, Epstein–Barr virus; LMP1, latent membrane protein 1; NF-κB, nuclear factor kappa B; CTAR/TES, C-terminal activating region/transformation effector site; TRAFs, tumour necrosis factor receptor-associated factors; Tregs, regulatory T cells; CCL20, C-C motif chemokine ligand 20; CTLs, cytotoxic T lymphocytes; NPC, nasopharyngeal cancer; miRNAs, microRNAs; UTR, untranslated region; IL-1R, interleukin-1 receptor; IL-1, interleukin-1).

In addition, it is postulated that aberrant NF-κB signalling and EBV latent genes may jointly contribute to the initiation and progression of NPC tumourigenesis by promoting tumour proliferation, survival, and immunosuppression (Figure 4) [89]. The C-terminal activating region or transformation effector site (CTAR/TES) of LMP1 can activate the canonical NF-κB pathway via interaction with the tumour necrosis factor receptor-associated factors (TRAFs), followed by phosphorylation of IκBα to sustain viral replication and persistent infection [40,90,91,92]. LMP1 is in turn upregulated by NF-κB, creating an amplification loop that results in constitutive NF-κB activity during EBV latency [93]. In addition, during lytic infection, BPLF1, an EBV deubiquitinase can deubiquitinate TRAF to antagonise NF-κB activity. Deubiquitination of TRAF can also downmodulate TLR signalling which acts as the first line defence in controlling viral infections [94,95]. As such, it impairs NF-κB activation and dampens pro-inflammatory cytokine processing in response to EBV, representing another strategy adopted by viruses to achieve immune evasion.

An upregulation of EBV BART is also associated with constitutive activation of NF-κB signalling in NPC cells [96,97], which provides a survival advantage and supports tumour development under stress conditions by upregulating expression of oncogenes and anti-apoptotic genes [92]. Inhibition of constitutive NF-κB activity has shown to suppress the proliferation and growth of EBV-positive NPC cells in vitro via downregulation of oncogenes and anti-apoptotic genes. Moreover, NF-κB-mediated expression of C-C motif chemokine ligand 20 (CCL20) in EBV-positive NPC cells was shown to protect infected cells from anti-viral immune response via recruitment of memory Tregs [89]. miR-BHRF1-2-5p inhibits NF-κB activation by triggering the downregulation of the IL-1 receptor 1 (IL-1R1), repressing NF-κB-mediated production of pro-inflammatory cytokines via inflammasome activation [98].

These findings suggest although NF-κB mediates the priming signal of inflammasome activation, upregulation of p62/SQSTM1 expression and NF-κB-induced mitochondria autophagy may diminish its pro-inflammatory function. Additionally, the persistent activation of NF-κB signalling may play a role in virus-mediated oncogenesis by enhancing its anti-apoptotic effects. Thus, NF-κB activation levels must be tightly regulated in NPC in order to elicit effective antitumour immune response.

### 3.4. Aberrant Production of Pro-Inflammatory Cytokines

The inflammasome activation also regulates and triggers inflammatory cytokines release. This molecular event underlines the immune response during viral infection. Multiple studies have demonstrated the important roles of IL-1β in early inflammation and activation of host antiviral response following viral infections [99,100]. Despite its essential role in mediating immune response against infected cells, the dysregulation of pro-inflammatory cytokines may become detrimental to the host by causing an excessive inflammation and providing a survival advantage for infected cells [100]. For instance, in healthy individuals, IL-1 signalling is tightly regulated by the expression of antagonists such as interleukin-1 receptor antagonist (IL-1Ra), to prevent unwanted tissue damage or excessive inflammation [98]. However, the IL-18 activation is related to its high affinity to IL-18 binding protein (IL-18BP) [101]. Viruses have developed multiple strategies to evade immune defences during infection, via manipulation of cytokine expression levels or by neutralising the immunological effects of cytokines.

The activation of IL-1β and IL-18 signalling may induce type I IFN response, which is essential in promoting apoptosis of infected cells. It has been demonstrated that EBV-encoded small RNAs (EBERs) can block IFN-α-mediated apoptosis by binding to RNA-activated protein kinase (PKR) and inhibiting its phosphorylation. PKR is a key mediator of the antiviral effect of IFN-α [102]. In addition, sequestration of IL-1β and IL-18 in the exosomes prevents them from binding to their cognate receptors, and hence inhibiting the activation of cytokine-induced type I IFN-mediated antiviral response while facilitating EBV persistence [103]. In fact, earlier study had reported that virus-infected cells could compromise host immune surveillance mechanism through intense sequestration of CC chemokines. This sequestration was reported to have a negative effect on the recruitment of leucocytes to the infection site, as well as suppressing the immune function of leucocytes in the infected cells [104]. As a result, virus-mediated sequestration of cytokines might be an additional mechanism for viruses to evade host immunity.

The upregulation of tumour inflammasome-derived IL-1β to a certain threshold has been shown to suppress the growth of EBV-associated NPC cells by promoting an influx of neutrophils, while depletion of these neutrophils significantly diminishes the antitumour effects of IL-1β [44]. A recent study has demonstrated that miR-BHRF1-2-5p, a EBV microRNA (miRNA) downregulates IL-1 receptor type 1 (IL-1R1) during EBV infection, altering the responsiveness of infected cells to IL-1β as well as downregulating the expression levels of IL-1β in EBV-infected cell populations [98]. EBV miR-BART15-3p directly targets the 3′ UTR of the NLRP3 inflammasome, consequently blocking cytokine production [71]. HPVs attenuate IL-1β production by facilitating proteasomal degradation of pro-IL-1β [80]. An upregulation of miR-197 by hepatitis B virus (HBV) significantly repressed IL-18 expression in the THP-1 cell line and positively correlated with the severity of HBV-associated liver disease symptoms, indicating towards a key role of IL-18 in modulating immune response against viral infections [105]. Similar to IL-1β, upregulation of IL-18 may facilitate tumour immune escape by recruiting MDSCs and promoting metastatic ability in tumour cells via upregulation of the vascular endothelial growth factor (VEGF) [21,106]. A study conducted in mouse models demonstrated that IL-18 upregulates surface expression of PD-1 on NK cells, thus subverting the antitumour effects of NK cells [107]. Similar findings are observed in NPC patient samples, in which increased levels of IL-18 appear to associate with poor prognosis and increase PD-1 expression on NK cell surface *in vitro*. These PD-1 expressing NK cells have shown to be functionally exhausted, diminishing their cytotoxicity against tumour cells [108].

Therefore, it is hypothesised that an imbalance of activators and antagonists may increase the severity of diseases. Indeed, excessive inflammation in IL-1Ra-deficient mice has been implicated in malignant transformation and carcinogenesis. An upregulation of IL-1β in IL-1Ra-deficient mice associates with an accumulation of MDSCs with enhanced immunosuppressive activity [109]. Cowpox viruses produce IL-1β receptor (vIL-1βR) and IL-18-BP to prevent their interaction with cellular receptors, thereby, blocking the downstream signalling of these cytokines [110]. Taken together, it is believed that disruption of cytokines signalling may affect not only host immune responses to viral infections, but may inadvertently contribute to the initiation and development of virus-associated carcinogenesis.

## 4. Epstein–Barr Virus (EBV)-Induced Sustained Inflammation in NPC Carcinogenesis

As discussed earlier, viruses may develop multiple strategies to avoid host inflammasome-mediated immune response. Paradoxically, viruses can also induce inflammasome activation to enhance its replication efficiency. Meanwhile, constitutive inflammasome activation may cause chronic inflammation, which is sustained by a high level of cytokine and chemokine expression, and consequently drives the development of tumour [111].

Over 90% of all humans would be infected with EBV during childhood. The primary infection is mostly asymptomatic, but associated with lifelong infection [112]. However, only a minority of the infected individuals would develop cancer, perhaps many years or even decades after the primary infection. Therefore, the progress from a primary infection to tumourigenesis is usually slow, and hence it requires additional oncogenic events, such as somatic mutation, immunosuppression, and exposure to carcinogens to catalyse tumourigenesis [113].

**Figure 5:**
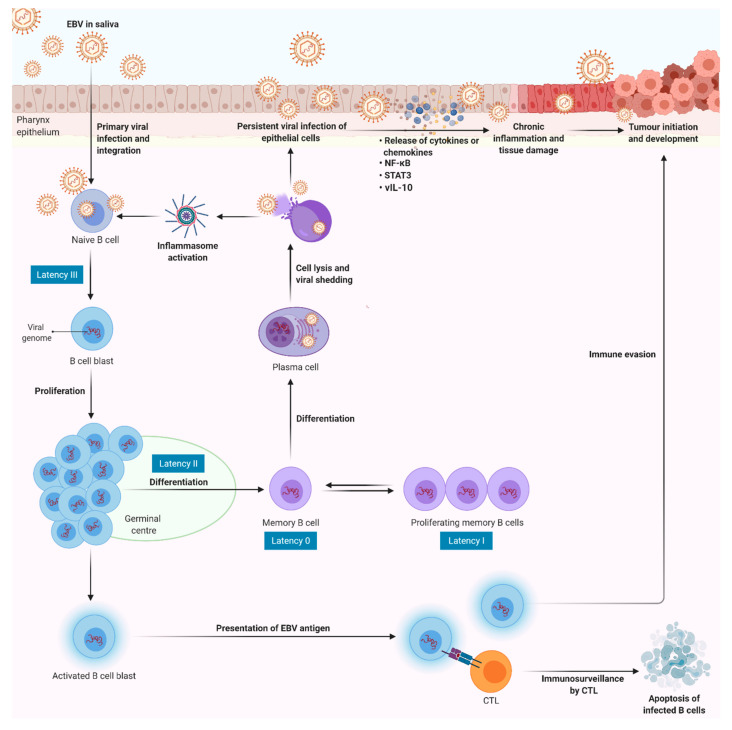
Overview of EBV-induced inflammation and NPC carcinogenesis. EBV in saliva passes through pharynx epithelium to infect naive B cells in the underlying lymphoid tissue via interaction of the viral glycoprotein gp350/220 with CD21 expressed on the B cells. This in turn drives the formation of proliferating lymphoblasts. Once EBV enters the cell, the viral capsid dissolves and viral genome incorporated into the host nucleus. The B cell blasts can differentiate into memory compartment as resting, long-lived, memory B cells with no viral gene expression. Furthermore, EBV encodes vIL-10 to escape host anti-viral responses and to establish latency by enhancing viability and differentiation of infected B cells. The latently infected memory B cells circulate in the periphery and return to the lymphoid tissue, where they can be signalled by cognate antigen to differentiate into plasma cells and activate viral replication program to produce more infectious virus. The lysis of infected cells leads the release of virion to initiate a new round of naïve B cell infection or infect the epithelial cells. Additionally, the plasma membrane break induces the efflux of potassium ion that activates inflammasome. Sustained inflammasome activation may promote viral replication and persistent infection. During a persistent infection, infected cells secrete excessive amount of pro-inflammatory cytokines/chemokines via NF-κB and STAT3 signalling, in order to establish a chronic inflammatory microenvironment that persistently damage the surrounding tissue. Over time, the chronic inflammation may trigger the initiation and development of cancer. In immunocompetent individuals, effective CTLs can recognise infected cells through the presentation of EBV antigen via MHC Class I and eliminate them by apoptosis. However, immune incompetent individual may be at risk of developing EBV-associated tumour during the chronic inflammation. (EBV, Epstein–Barr virus; NF-κB, nuclear factor kappa B; STAT3; signal transducer and activator of transcription 3; CTLs, cytotoxic T lymphocytes; MHC, major histocompatibility complex; vIL-10, viral IL-10).

EBV is transmitted through the saliva, entering the lymphoid tissue of Waldeyer’s ring and infecting the naive B cells (Latency III) [114]. Infection of B cells by EBV is initiated through the interaction of viral glycoprotein, gp350/220, with the complement receptor expressed on the B cell surface, CD21 [115]. When the naive B cells detect cognate antigen, they become activated into a proliferating lymphoblasts. Activated blasts enter a the germinal centre (GC) reaction in Latency II, in which LMP1 and LMP2 can provide surrogate antigen and Th survival signals [114]. This enables the latently infected B cells to leave the GC and enter into memory compartment as resting, long-lived, memory B cells. In this setting, the virus maintains a latent state as an episome and does not express any viral genes (Latency 0), thereby allowing infected memory B cells to completely evade immune surveillance [116]. Latently infected memory B cells circulate in the periphery and return to the lymphoid tissue, where they can be signalled by cognate antigen and differentiate into plasma cells, enabling viral replication and release of infectious virus. The released virus can initiate a new round of naive B cell infection or infect the epithelium [114,117]. Taken together, resting B cells serve as an important reservoir to infect epithelial cells, while the epithelium is the main site for lytic infection, producing more infectious virus for cell to cell spreading and transmission of virus to new hosts [116,118]. Needless to say, viral antigens expressed by the infected cells represent the primary target for immune detection. In response to the viral infection, infected individuals will develop a strong immune response with the help of NK cells and CTLs. However, when the viral infection is neither cleared nor eliminated from the body, the surviving virus will progress to evade the host’s immune response [119]. It is hypothesised that EBV evades host innate and adaptive immune responses, perhaps through immunosuppression or interference with inflammation (Figure 5).

The functional relationship between inflammation and tumour progression has been discussed since year 1863 [120]. In general, inflammation is tightly regulated by physiological processes in response to a cellular injury or infection. Acute inflammatory response is important in initiating antiviral response by diverse populations of leucocytes, while excessive inflammation that arisen during persistent infection has been shown to contribute significantly to tumour formation and metastasis [121]. It has been reported that EBV constitutively activates the inflammasome to promote viral replication in the host [122]. Moreover, EBV can initiate carcinogenesis by inducing chronic inflammation via NF-κB and signal transducer and activator of transcription 3 (STAT3) signalling to create a chronic inflammatory microenvironment that supports cancer development [121]. Previous study has demonstrated the role of EBERs as a strong inducer of chronic inflammation, driving the transition of inflammation to oncogenesis in the nasopharynx via the TLR-3 pathway. EBER-induced activation of the TLR-3 pathway promotes the production of pro-inflammatory cytokines via NF-κB. LMP1 also stimulates NF-κB to generate proliferative signals in EBV-infected cells [123]. Furthermore, EBV has been shown to suppress the immune response during its lytic phase by inducing the production of cellular cytokines, such as IL-8 and IL-10 to support the maturation and release of progeny virions [124,125,126]. Lytic proteins, such as Z transactivator (Zta, also known as BZLF1) and BCRF1, are identified as the important cytokine mediators. For example, the product of EBV-BCRF1, viral IL-10 (vIL-10) reduces the expression of MHC Class I/II molecules and intercellular adhesion molecule 1 (ICAM1), impeding T cell activation. ICAM1 is an adhesion molecule which is essential for the interaction of antigen presenting cells (APC) with T cells [127]. Multiple studies have reported that BCRF1 may exert protective effect against immune surveillance during the lytic cycle through the inhibition of IFN-γ production by NK cells and T cells, and ultimately enhancing the survival of infected B cells as well as dissemination of EBV [128,129]. On the other hand, the viral transcription factor, BZLF1 acts as a molecular switch to drive the transition from the latent infection to lytic cycle, producing new virions [130]. Viral Zta protein has been shown to promote NPC progression by increasing the expression of matrix metalloproteinase (MMP) 1 and 9 to enhance cell invasiveness [131,132]. EBV lytic phase has also been shown to aid in immune evasion by preventing viral antigen presentation through MHC molecules. BNLF2a, an immune evasion protein encoded by EBV is expressed during early lytic phase replication. Ectopic expression of BNLF2a leads to the downregulation of MHC Class I molecules, inhibiting the presentation of antigen to CTLs and elimination of EBV-infected cells. Hence, it allows the production of more viral progeny [133,134]. Therefore, it is believed that EBV may interfere with inflammatory signalling pathways to support its persistent infection while subverting the resulting inflammatory responses to avoid immune recognition and elimination, driving the development of NPC.

## 5. Roles of Inflammasome in NPC and Other Malignancies

Inflammasome activation is important in suppressing tumour progression via secretion of pro-inflammatory cytokines and inducing pyroptosis. For example, secretion of IL-1β during acute inflammation demonstrates a beneficial role in the clearance of infections by promoting the expansion of NK cells as well as CD4^+^ T cells and CTLs [135]. Therefore, it is hypothesised that if the tumour cells fail to elicit inflammasome-mediated immune response, they would be recognised as self and will then escape from T cell-mediated immune surveillance, which further enhances tumour progression. However, inflammasomes have been shown to have contrasting roles in carcinogenesis (Table 1).

Overexpression of the NLRP1 inflammasome was found to enhance breast cancer cell proliferation, metastasis, and tumourigenicity in nude mice by promoting epithelial-mesenchymal transition (EMT) with the downregulation of E-cadherin and upregulation of mesenchymal markers, such as vimentin, matrix metalloproteinase (MMP)-9, and Snail [138]. In contrast, knockdown of the NLRP3 inflammasome in oral squamous cell carcinoma (OSCC) cells significantly suppressed tumour proliferation and was associated with an upregulation of E-cadherin, indicating that inflammasome activation is involved in promoting metastasis via EMT during carcinogenesis [136]. Similar results have also been reported in lung cancer [141] and colon cancer [143]. In head and neck squamous cell carcinoma (HNSCC), overexpression and constitutive activation of the NLRP3 inflammasome was closely correlated with carcinogenesis and enhanced self-renewal capacity of cancer stem cells [146]. Aberrant NLRP3 inflammasome activation induces the overproduction of pro-inflammatory cytokines to form an inflammatory microenvironment and promotes tumour progression by reducing the drug sensitivity of tumour cells [146].

Intriguingly, overexpression of the NLRP3 inflammasome in EBV-associated NPC was shown to associate with favourable clinical outcome, suggesting the role of the inflammasome in tumour suppression [44]. Furthermore, the administration of tumour-derived IL-1β into tumour-bearing mice was shown to significantly suppress tumour growth by inducing an influx of tumour-associated neutrophils (TANs) to the tumour site, while the decrease of TANs significantly abrogated IL-1β-mediated antitumour activity [44]. Together, these findings suggest that inflammasomes may attenuate tumour progression through IL-1β production and recruitment of TANs. However, some studies show that elevated expression of IL-1β would contribute to chronic inflammation that promotes tumour development and immunosuppression. For example, overproduction of IL-1β resulting from constitutive NLRP3 inflammasome activation caused auto-inflammation and enhanced tumour progression in melanoma [137]. In pancreatic cancer, ATP-induced NLRP3 inflammasome activation accelerates IL-1β production that subsequently increases tumour cell proliferation [148]. Upregulation of the NLRP3 inflammasome in OSCC cells was reported to correlate positively with IL-1β expression level, tumour growth, and lymph node metastatic status [136]. Tumour promoting effect of the NLRP3 inflammasome can be reversed by downregulating its expression levels or pharmacological inhibiting their activity. Pharmacological inhibition of the NLRP3 inflammasome was shown to restore innate and adaptive antitumour immune response, including CTL activation and polarisation of Th1 cells [149]. Similarly, inflammasome-derived IL-18 also demonstrated dual effects in tumour progression. Dagenais and Saleh indicated that inflammasome-derived IL-18 is essential for the priming of NK cells and subsequent triggering of FasL-induced apoptosis [144]. Furthermore, it has been shown that inflammasome-deficient mice produced markedly reduced levels of IL-18, which was concomitant with impaired production and activation of tumour suppressor genes IFN-γ and STAT1 [145]. Conversely, an increased in NLRP1 inflammasome-mediated IL-18 production was found to induce immunosuppression in multiple myeloma tumour-bearing mice by promoting recruitment of MDSCs and hampering the cytotoxic effects of CTLs [21].

Caspase-1, a key player of pyroptosis, has been shown to act as a tumour suppressor in carcinogenesis. A recent study demonstrated that the downregulation of caspase-1 in breast cancer cells was significantly associated with increased tumour proliferation and invasion, and decreased apoptosis [140]. In lung cancer, caspase-1 inhibition results in the reduction of IL-1β and IL-18, leading to tumour-induced immunosuppression in the lung microenvironment [142]. In contrast, inhibition of caspase-1 enhances immune surveillance in pancreatic cancer by reducing Treg populations while promoting CTL activation [149]. Constitutive activation of caspase-1 in EBV-associated cancer facilitates viral genome persistence and immune evasion via cleavage of the pro-forms of IL-1β and IL-18 [103]. Therefore, caspase-1-mediated programmed cell death may remove tumour cells and suppress tumour progression, but improper and excessive activation may promote carcinogenesis through suppression of immune surveillance.

P2X7 plays an important role in activating the NLRP3 inflammasome. ATP released from the dying tumour cells would bind to the P2X7R expressed on dendritic cells (DCs) to activate the inflammasome, allowing for the production of IL-1β and activation of CTLs [50]. Hence, the loss of functional P2X7R would lower its affinity for ATP, blocking the activation of the NLRP3 inflammasome. As a consequence, P2X7-deficient mice result in the suppression of CTL-mediated antitumour response and are associated with enhanced metastatic capacity [50]. However, inhibition of both the NLRP3 inflammasome and P2X7R significantly reduced invasiveness and viability of HNSCC cells [147].

In colitis-associated cancer (CAC) and colorectal cancer (CRC), the inflammasome acts as a tumour suppressor to protect against tumour development. NLRP1-, NLRP3-, and caspase-1-deficient mice were found to be highly susceptible to dextran sulphate sodium/azoxymethane (DSS/AOM)-induced CAC. These mice suffered from increased tumour burden and attenuated levels of IL-1β and IL-18, indicating that the inflammasome and its downstream cytokines play a protective role against CAC [150,151]. Moreover, decreased production of IL-18 in NLRP3- and caspase-1-deficient mice was associated with significantly higher mortality rates. These mice were more vulnerable to DSS-induced epithelial barrier injury in the gut compared to DSS-fed wild-type mice, and suffered from decreased barrier function and the loss of epithelial integrity, causing an overgrowth of commensal microflora, massive infiltration of leucocytes and increased chemokine production in the colon. All of these outcomes could further exacerbate disease severity [152]. These findings highlight the important roles of the NLRP3 inflammasome and IL-18 in the regulation of intestinal homeostasis. In addition, the production of NLRP3-mediated IL-18 was shown to promote the maturation of NK cells and enhance immune surveillance by suppressing liver metastatic growth of CRC cells in mouse models [49]. Conversely, the markedly reduced IL-18 expression in NLRP3-deficient mice results in dramatically increased tumour burden in the colon [145]. Hence, it is suggested that the increased tumour growth resulting from inflammasome impairment might be due to the dampened antitumour immune response, which is mediated by inflammasome-induced cytokines that in turn recruit immune effector cells such as DCs, NK cells, and T cells.

In summary, the findings discussed above show that inflammasome components and their downstream cytokines play complicated roles in the initiation, progression, and regression of tumour. The tumour promoting and suppressive effect of inflammasome appears to be dependent on the context and tumour types.

## 6. Therapeutic Potential of the Inflammasomes in Cancer Immunotherapy

Chronic inflammation as a result of persistent viral infection is increasingly being recognised as a driver of tumour initiation and development. The contrasting roles of the inflammasomes and its downstream cytokines have gained attention in cancer development and cancer therapy. Targeting the inflammasomes may provide a promising opportunity for cancer prevention, either by controlling viral replication or preventing excessive virus-induced inflammation. In addition, drugs or inhibitors targeting inflammatory signalling pathways, such as NF-κB utilised by oncogenic viruses to establish persistent infections is expected to reduce the incidence of virus-mediated cancer, overcome tumour resistance, and enhance the synergistic effect of combination therapy [93,153]. Recently, cancer immunotherapy has evolved as a means to enhance antitumour response and therapeutic efficiency through increasing tumour antigenicity, reprogramming of TME, and systemic inhibition of immunosuppressive cells to checkpoint blockades [154].

However, a recently concluded clinical trial (NCT02339558) using nivolumab, a highly selective humanised monoclonal antibody against PD-1 for treatment of recurrent and metastatic NPC demonstrated a relatively low objective response rate of only 20.5% [155]. In the phase Ib KEYNOTE-001 trial (NCT01295827), pembrolizumab, an anti-PD-1 antibody demonstrated antitumor activity against melanoma with overall response rate of 41% [156]. In contrast, only 25.9% of overall response rate was reported in a phase Ib KEYNOTE-028 trial (NCT02054806) using pembrolizumab for patients with recurrent and metastatic NPC [157]. A recent study also revealed that NLRP3 is a promising pharmacological target for enhancing the therapeutic efficacy of immune checkpoint inhibitor. Genetic silencing and pharmacological inhibition of NLRP3 inflammasome activation was shown to significantly enhance the efficacy of anti-PD-1 immunotherapy by diminishing the infiltration of MDSCs [158].

As inflammasome activation plays a role in altering the expression levels of pro-inflammatory cytokines and differentiation of immune effector cells, targeted inhibition of inflammasome activation may restore the effective immune surveillance by augmenting antitumour immunity [42]. Development of inhibitors to suppress overactivated IL-1β signalling activity represents one of the cancer therapeutic strategies for inflammasome inhibition. These IL-1β signalling inhibitors include monoclonal antibodies and recombinant IL-1 receptor (IL-1R) antagonists, which neutralise IL-1β and decrease IL-1 activity, respectively [20]. In vivo, an impressive synergistic effect was shown with the combination of anti-IL-1β and anti-PD-1, suggesting that IL-1β neutralisation may reduce inflammation-mediated immunosuppression and enhance responsiveness to immune checkpoint inhibitors [159].

Canakinumab, a human anti-IL-1β monoclonal antibody has been approved as the first line treatment by the U.S. Food and Drug Administration (FDA) for inflammasome-related diseases [160]. The antitumour efficiency of canakinumab has been determined by Canakinumab Anti-inflammatory Thrombosis Outcomes Study (CANTOS) involving over 10,000 atherosclerosis patients who had had a myocardial infarction (NCT01327846). Compared to the placebo group, canakinumab-receiving patients were associated with a lower rate of recurrent vascular events and a significant reduction in incidence and mortality rates of lung cancer. These data suggest that canakinumab, which targets the inflammasome-derived IL-1β, may reduce inflammation that could progress into cancer [161,162]. Multiple clinical trials of canakinumab in combination with immune checkpoint inhibitors are ongoing. For example, a phase II clinical trial (NCT03484923) is ongoing to evaluate the safety and efficacy of canakinumab with anti-PD-1 (PD001/spartalizumab) in previously treated unresectable or metastatic melanoma patients. Another phase I clinical trial (NCT02900664) is aiming to determine the safety, tolerability, pharmacological and clinical activity of canakinumab with PD001 in CRC, triple negative breast cancer, and non-small cell lung cancer (NSCLC). Currently, the application of canakinumab has not been focused on NPC but is theorised to combat NPC in a similar way–by preventing excessive inflammation due to its anti-inflammatory activity.

Anakinra, the first recombinant IL-1R antagonist was approved by the US Food and Drug Administration (FDA) in year 2001 to treat rheumatoid arthritis [163]. Anakinra can inhibit the binding of IL-1β and IL-1R and is widely used as adjunct therapy to lower IL-1-driven inflammation and immunosuppression in cancer. Treatment of HER2-negative breast cancer with anakinra plus chemotherapeutic drugs (NCT01802970) was shown to reduce inflammation and restore cytotoxicity of NK cells and CTLs, hence enhancing the clinical efficacy of checkpoint blockades [164,165,166]. In a mouse xenograft model of erlotinib-resistant HNSCC, IL-1 blockade by anakinra could overcome erlotinib resistance and significantly reduce the circulating levels of IL-1β. This in turn dampens the inflammatory TME and improves drug response, suppressing tumour growth [167]. IL-18 blockade in osteosarcoma cancer was shown to inhibit MDSC recruitment and suppress tumour growth. Combination treatment of IL-18 blockade with anti-PD-1 significantly improved the efficacy of immune checkpoint inhibitors by promoting infiltration of CTLs and production of granzyme B and IFN-γ [168].

Oligomerisation of ASC is essential in activating inflammasomes and subsequent induction of cytokines production. Hence, efficient blocking of ASC oligomerisation may represent another potential therapeutic target in the treatment of cancer. The discovery of MCC950, a selective inhibitor of the NLRP3 inflammasome, has been shown to remarkably reduce IL-1β production and infiltration of immunosuppressive cells, such as MDSCs, TAMs, and Tregs in the TME of HNSCC mouse model [169]. The number of effective CD4^+^ T cells and CTLs were significantly increased, and the PD-1/TIM-3-positive T cells significantly reduced in MCC950-treated HNSCC mouse. Expression of PD-1/TIM-3 on T cells is known to impair effector function of T cells, leading to T cell exhaustion. Hence, these findings indicate that blocking NLRP3 inflammasome activation with MCC950 may delay tumour growth and enhance checkpoint inhibitors responsiveness through the upregulation of effector antitumour T cells [169], clearly highlighting the usefulness of MCC950 as a potent therapeutic in inflammasome-associated cancers.

In summary, these data indicate that the inhibition of inflammasomes activity may have a preventive and therapeutic potential in reducing or preventing excessive inflammatory responses at the tumour site. At the same time, it also helps to boost the clinical efficacy of immune checkpoint inhibitors in cancer treatment by inhibiting the infiltration of immunosuppressive cells and hence restraining tumour growth and progression.

## 7. Roles of Other Inflammasome Inhibitors in Cancer Therapy

Other possibilities to regulate production of pro-inflammatory cytokines include targeting of the inflammasome itself and its components. In vitro, administration of Ac-YVAD-CHO, a caspase-1 inhibitor into NPC cell lines was shown to significantly suppress the secretion of IL-18, indicating that IL-18 production is caspase-1 dependent [170]. The upregulation of tumour-derived IL-18 may drive the massive infiltration of leucocytes but fail to halt the tumour growth. The interaction of CD40 expressed on NPC cell surface with CD40L on infiltrating T cells results in strongly inhibition of Fas-mediated cytotoxicity, thereby protecting the NPC cells from immune surveillance [170]. Belnacasan (VX-765) and pralnacasan (VX-740) are also served as caspase-1 inhibitors, downregulating caspase-1 activity and inhibiting the subsequent release of IL-1β and IL-18 in inflammatory diseases [45]. Belnacasan has also been shown to reduce lung inflammation in mouse model by suppressing the NLRP3 inflammasome and caspase-1 activity, leading to a decrease in cytokine production [171]. In DSS-induced murine colitis models, pralnacasan significantly attenuated DSS-induced colitis by suppressing pro-inflammatory cytokine activation [172]. An immunomodulator, known as thalidomide, exerts its anti-inflammatory effect via inhibition of caspase-1 activation and IL-1β production. Disruption of inflammasome activation and subsequent caspase-1 activation by thalidomide has shown to suppress tumour growth in relapsed myeloma patients through its anti-angiogenic activity [173].

Parthenolide is a naturally occurring sesquiterpene lactone compound that found in the feverfew plant. It is widely used as an anti-inflammatory drug that inhibits NF-κB activity via interaction with IKK or directly with p65 subunit, resulting in the inhibition of the transcription of anti-apoptotic genes [174]. In vitro, parthenolide exerts its tumour suppressive effect in CRC by suppressing the expression of migration/invasion-related proteins, such as E-cadherin, Snail, MMP-2, MMP-9, and cyclooxygenase-2 (COX-2) [175]. In addition, parthenolide has been shown to greatly sensitise a variety of tumour cells, including NPC to TNF-α-induced apoptosis by preventing constitutive activation of NF-κB in tumour cells [176]. Additionally, combination regimen of parthenolide with cyclooxygenase-2 (COX-2) inhibitors markedly inhibited proliferation and enhanced apoptosis sensitivity of NPC cells [177]. Similarly, the antitumour effects of parthenolide was significantly enhanced when it was used in combination with ganciclovir (GCV), an antiviral drug for the treatment of Burkitt lymphoma [178]. Balsalazide is an azo bond compound of 5-aminoslaicylic acid (5-ASA) and 4-aminobenzoyl-β-alanine used in the treatment of inflammatory bowel disease. It is effective in triggering anti-inflammatory response via downregulation of NF-κB and IL-1 [179]. Surprisingly, parthenolide and balsalazide synergistically suppressed tumour proliferation and induced apoptosis in CRC cells by inhibiting nuclear translocation of p65 subunit and downregulation of anti-apoptotic protein expression [179,180]. As a result, parthenolide monotherapy could be useful in regulating inflammatory response or in combination with other therapeutic agents to synergistically suppress tumour progression.

Andrographolide is a labdane diterpenoid that is extracted from the stem and leaves of *Andrographis paniculata*, which has a broad range of pharmacological properties, such as antibacterial, antiviral, anti-inflammatory, antitumour, and so on [181]. As an anti-inflammatory agent, it reduces the expression levels of pro-inflammatory cytokines and inflammasomes activation via downregulation of the NLRP3 inflammasome and caspase-1 [181]. It has also been shown to suppress proliferation and invasion of NPC cells by blocking constitutive activation of NF-κB that favours the development of inflammatory and pro-survival TME to promote and sustain tumour survival [182]. In vitro, treatment of NPC cells with andrographolide significantly induced cell cycle arrest in G2/M phase and apoptotic cell death. Furthermore, expression of invasion and metastasis-related genes such as MMP-9, VEGF, and intercellular adhesion molecule (ICAM)-1 were downregulated in NPC cells after andrographolide treatment [182]. As a result, these findings clearly demonstrate the role of andrographolide as a potential NF-κB inhibitor as well as an anti-cancer agent in NPC. Moreover, andrographolide prevents colitis-associated tumourigenesis by triggering mitophagy in the macrophages of colitis-associated tumour-bearing mice. Consequently, ATP, which acts as an inflammasome activator, cannot be released from the damaged mitochondria, resulting in suppressed NLRP3 inflammasome activation and reduced IL-1β secretion [183]. However, these findings are in contrast with CAC studies in NLRP3 inflammasome-, ASC-, or caspase-1-deficient tumour models. ASC- and caspase-1-deficient mice demonstrate increased disease severity due to the attenuated levels of IL-1β and IL-18 [150]. Hence, it is suggested that a complete knockout of inflammasome components may lead to imbalance cytokines/chemokines profile in the TME, which could promote aggressiveness of the diseases.

## 8. Conclusions and Future Perspective

Inflammasomes activation and subsequent inflammatory response play an essential role in eradicating viral infections as well as suppressing tumour growth. However, dysregulation of inflammasomes signalling and subsequent aberrant production of pro-inflammatory cytokines have led to chronic inflammation, which drives viral replication and tumour progression. Targeted inhibition of inflammasome activity may have potential applications in cancer prevention and therapy due to its inhibitory activity against ongoing inflammation, which may increase the severity of diseases, and by providing an opportunity to improve the efficiency of the immune checkpoint blockade in cancer immunotherapy. Recently, studies have elucidated several strategies used by viruses to evade inflammasome-mediated antitumour immune response. However, the evasion mechanisms in EBV-associated cancers are far from fully elucidated. Additionally, there is little reported on the clinical role of NLR inflammasomes in response to EBV infection. The contrasting effects of inflammasomes in cancer further suggest that there is much about the functional roles and mechanisms involved that remain to be determined. Therefore, additional investigations are required to reveal the functional roles and mechanisms underlying the activation of the inflammasomes in NPC. These findings will be useful for designing more specific and effective therapeutic agents for the treatment of NPC. Moreover, further pharmacological studies and clinical trials are warranted to determine the safety and efficacy of therapeutic agents targeting at the inflammasome complexes.

## Figures and Tables

**Table 1:** The contrasting roles of inflammasomes in cancer.

Tumour Types	Roles of Inflammasomes	Inflammasomes and Signalling Molecules		Results	Reference
NPC	Tumour suppressor	NLRP3, IL-1β		Upregulation tumour inflammasome-derived IL-1β actively induced the influx of TANs to the tumour site and positively correlated to patient survival.	[44]
	Tumour promoter	Caspase-1, IL-1β, IL-18		Constitutive activation of caspase-1 facilitated EBV genome persistence and immune evasion.	[103]
Multiple myeloma	Tumour promoter	NLRP1, IL-18		NLRP1-mediated IL-18 induced immunosuppression through MDSC recruitment.	[21]
OSCC	Tumour promoter	NLRP3		Knockdown of NLRP3 increased E-cadherin expression and suppressed proliferation, invasion, and migration of OSCC cells.	[136]
Melanoma	Tumour promoter	NLRP3, IL-1β		NLRP3 activation increased IL-1β secretion, resulting in auto-inflammation and enhanced tumour growth.	[137]
Breast cancer	Tumour promoter	NLRP1, IL-1β, IL-18	NLRP1 promoted tumour progression by inducing EMT, both in vivo and in vitro.		[138]
	Tumour promoter	NLRP3, IL-1β	NLRP3 activation and IL-1β promoted tumour growth and lung metastasis via increased infiltration of MDSCs and TAMs.		[139]
	Tumour suppressor	Caspase-1	Downregulation of caspase-1 increased breast cancer cell proliferation and invasion.		[140]
Lung cancer	Tumour promoter	NLRP3	NLRP3 activation decreased E-cadherin expression and promoted proliferation and migration of lung cancer cells.		[141]
	Tumour suppressor	NLRP3, Caspase-1	Impairment of NLRP3/caspase-1 activation promoted immunosuppression in the lung microenvironment.		[142]
CRC	Tumour promoter	NLRP3	Knockdown of NLRP3 reversed mesenchymal phenotypes of tumour cells and diminished tumour migration and invasion.		[143]
	Tumour suppressor	NLRP3, IL-18	NLRP3-derived IL-18 enhanced NK cell priming and trigger FasL-induced apoptosis of CRC cells.		[144]
	Tumour suppressor	NLRP3, IL-18	NLRP3 activation enhanced maturation and tumouricidal activity of NK cells as well as suppressed metastatic growth of CRC in liver.		[49,145]
HNSCC	Tumour promoter	NLRP3, IL-1β	Upregulation of NLRP3 activity positively correlated to carcinogenesis and CSC markers.		[146]
	-	NLRP3, P2X7R	Blockade of NLRP3 activation and P2X7R significantly reduced invasiveness and viability of HNSCC cells.		[147]
Pancreatic cancer	Tumour promoter	NLRP3, caspase-1, IL-1β	NLRP3 activation promoted tumour survival via upregulation of caspase-1 activity and IL-1β production.		[148]
	-	NLRP3, ASC, caspase-1	Inhibition of NLRP3, ASC, or caspase-1 activation restored innate and adaptive antitumour immune response.		[149]
CAC	Tumour suppressor	NLRP1, NLRP3, IL-1β, IL-18	Loss of inflammasome-secreted IL-1β and IL-18 increased susceptibility of mice to CAC.		[150,151]
	-	NLRP3, IL-18	Defective NLRP3 activation decreased IL-18 production and led to the overgrowth of commensal, massive infiltration of leucocytes, and overproduction of chemokines in the colon, which ultimately, resulted in increased mortality rates.		[152]

NPC, nasopharyngeal cancer; NLRP3, NOD-like receptor pyrin domain containing 3; IL-1β, interleukin-1β; TANs, tumour-associated neutrophils; IL-18, interleukin-18; EBV, Epstein–Barr virus; NLRP1, NOD-like receptor pyrin domain containing 1; MDSCs, myeloid-derived suppressor cells; OSCC, oral squamous cell carcinoma; EMT, epithelial-mesenchymal transition; TAMs, tumour-associated macrophages; CRC, colorectal cancer; NK cells, natural killer cells; HNSCC, head and neck squamous cell carcinoma; CSC, cancer stem cell; P2X7R, P2X7 purinergic receptor; ASC, apoptosis-associated speck-like protein; CAC, colitis-associated cancer.

## Data Availability

Data sharing not applicable.
